# Audit on patient outcome based on APACHE II scoring in the respiratory ICU of a south Indian university teaching hospital

**DOI:** 10.1186/cc11750

**Published:** 2012-11-14

**Authors:** H Mangu, M Anakapalli, A Samantaray

**Affiliations:** 1 Sri Venkateswara Institute of Medical Sciences, Tirupati, India

## Background

The Acute Physiology and Chronic Health Evaluation (APACHE II) score is widely used in the ICU and has been well validated against the other critical illness scoring systems in predicting ICU mortality. The aim of this audit was to determine survival to hospital discharge for patients admitted to our ICU during a period of 6 months.

## Methods

We performed a prospective audit of 80 consecutive patients admitted to our 16-bed respiratory intensive care unit (RICU) in a university teaching hospital. Patients were excluded if the observation period was less than 24 hours and age less than 12 years. An audit form was completed by a fellow intensivist who followed up the patients until they were discharged from the RICU. Patient data were stratified according to the outcome (S, survivors; E, expired) and compared for different age groups (12 to 29 years, 30 to 60 years, >60 years), gender (males vs. females) and diagnostic disease groups. Student's *t *test, chi-square test/Fisher's exact test and analysis of variance were used for comparison of data as appropriate and *P *< 0.05 was considered significant.

## Results

The mean APACHE II score, mean age and duration of ICU stay were 12.4 ± 10.8, 42.1 ± 20.6 years and 12.8 ± 12.2 days respectively. The patients' number of days of ICU stay was independent of patients' outcome (S or E) (*P *= 0.476). Similarly the gender distribution did not affect the mean APACHE II score on admission (*P *= 0.273) and duration of ICU stay (*P *= 0.166). The survival rate among the eight different diagnostic groups was similar (*P *= 0.064). A higher APACHE II score and a higher age was associated with increased mortality (*P *< 0.001 and *P *= 0.001 respectively).

## Conclusion

Our RICU had a mortality of 32% (26/80 patients) and patients who died belonged to the higher age group and had high mean APACHE II score. Although our audit is small and may not represent the cohort adequately, our mortality rate was similar to that obtained from a large cohort [1]. The higher mortality rate was probably because our centre is a tertiary-care referral hospital and many referred cases reached us late.
